# Comparison of blood parameters in two genetically different groups of horses for functional longevity in show jumping

**DOI:** 10.3389/fgene.2024.1455790

**Published:** 2024-10-29

**Authors:** Suzanne Harari, Severine Deretz, Bernard Dumont Saint Priest, Eric Richard, Anne Ricard

**Affiliations:** ^1^ Université Paris Saclay, Institut National de Recherche Pour l'Agriculture, l'Alimentation et l'Environnement, AgroParisTech, Génétique Animale et Biologie Intégrative, Jouy-en-Josas, France; ^2^ Institut Français du Cheval et de l’Equitation, Pôle développement, innovation et recherche, Saumur, France; ^3^ EA 7450 BIOTARGEN, Université de Caen Normandie, Caen, France

**Keywords:** show jumping horses, blood parameters, functional longevity, animal genetics, heritability, early criteria, animal welfare

## Abstract

**Introduction:**

In order to find early selection criteria to improve the longevity of show jumping horses, a specific protocol was designed.

**Methods:**

Before entering competition, young horses selected from extreme stallions for longevity were measured for many traits, including blood parameters. Blood samples were taken from 952 horses aged 2–4 years old, sired by two groups of stallions: one with unfavorable (U) and the other with favorable (F) extreme estimated breeding values for functional longevity. These breeding values were previously calculated from data on 202,320 horses that participated in show jumping competitions between 1985 and 2022. Functional longevity was defined as time spent in competition, adjusted for the level of performance. The 59 measured parameters included hematology, proteins, cytokines, liver and kidney function, bone and joint health, oxidative stress and endocrinology. Heritability was estimated using a mixed model that accounted for the effect of age, sex, estimated weight, visit (place and date of collection), and animal random additive value with 10,280 horses in pedigree. A Partial Least Square logistic regression was performed to predict the sire group.

**Results:**

Age, sex and estimated weight significantly affected 36, 19 and 16 variables, respectively. The visit had a significant effect on all variables. Heritability estimates were high, with 75% higher than 0.20% and 30% higher than 0.50. The most heritable traits included mean corpuscular volume (0.92, se 0.11), mean corpuscular hemoglobin (0.90, se 0.11), white blood cells (0.55, se 0.13), total alkaline phosphatase (0.68, se 0.12) and percentage of γ-globulin (0.57, se 0.12). The logistic regression that predicted the group of sires favorable for longevity identified 16 significant variables. Key findings included: lower mean corpuscular hemoglobin (*p*-value < 0.001), lower mean corpuscular volume (*p*-value < 0.001), lower number of white blood cells (*p*-value < 0.01), higher percentage of intestinal and bone alkaline phosphatase (*p*-value < 0.01) for a lower total alkaline phosphatase (*p*-value < 0.01), higher percentage of α2-globulin (*p*-value < 0.001) and lower percentage of β1-globulin (*p*-value < 0.01).

**Discussion:**

Blood parameters measured at rest in young horses may be predictive of their genetic value for functional longevity in show jumping.

## 1 Introduction

Show jumping is one of the three Olympic equestrian sports. In addition to representing significant economic stakes, show jumping competitions also raise concerns about animal welfare, particularly due to the risks of injuries. Breeding organizations for sport horses now include these considerations in their selection objectives (Hartig et al., 2013; Koenen et al., 2004). Several authors have previously studied longevity for sport horses (Braam et al., 2011; Posta et al., 2014; Ricard and Blouin, 2011; Seierø et al., 2016; Solé et al., 2017; Wallin et al., 2000), the main aims being the identification of the potential factors of risks and causes of culling. The functional longevity is defined by the longevity in active life, i.e., in competition, corrected for the level of performance. It is an important indicator of a show jumping horse’s wellbeing. Indeed, improving functional longevity means enhancing the wellbeing of horses in competition and the physical resistance of the horse. However, selection on the basis on the estimated breeding value calculated from competition data has its limits. In France, the heritability of this trait is low (0.12 in Dugué et al., 2021). At the optimal age for selection, around 5 years (Dubois et al., 2008), own competition lifespan is likely to be censored, and thus less informative. Therefore, the reliability of the estimated breeding value for functional longevity based on own and relatives results in competition, remains low. To be effective in terms of selection and to improve the health and wellbeing of sport horses, breeding evaluations must be conducted earlier and with greater accuracy than those based solely on competition results. In order to do this, several teams have sought early indicators of longevity, but these studies have encountered several limitations. Firstly, some have only examined observed longevity rather than functional longevity, resulting in confusion between traits related to performance and those associated with functional longevity (e.g., Jönsson et al., 2014; Seierø et al., 2016). In fact, performance is the primary factor influencing observed longevity. Secondly, the indirect traits usually used are those measured from young horse field tests - e.g., conformation, linear traits and gaits (Jönsson et al., 2014; Seierø et al., 2016; Wallin et al., 2001) - and are merely related to performance and thus to observed longevity. Consequently, there are currently no measured traits that may be directly associated with functional longevity. This highlights the need for innovative approaches; this is why a new and specific protocol had to be developed.

However, some traits related to general conformation, orthopedic status and locomotor health have occasionally been identified (Ducro et al., 2009; Jönsson et al., 2014; Seierø et al., 2016; Stock and Distl, 2005; Wallin et al., 2001).

Our study aimed to identify early and innovative criteria for predicting the genetic value for functional longevity, which could be useful in selection breeding plans. To achieve this, we built a specific protocol based on divergent selection of stallions on breeding values for functional longevity and measured the early criteria on their progeny before they enter competition. This paper reports on the design of the protocol and results for blood parameters.

## 2 Materials and methods

### 2.1 Description of protocol

The protocol aimed to establish a high genetic contrast for functional longevity between two groups of horses and to assess various indirect traits potentially related to functional longevity before they began competing. To achieve this, we used breeding values for functional longevity. The Estimated Breeding Value (EBV) of stallions for functional longevity in jumping competition was obtained from the analysis of competition data from 1985 to 2022. This competition dataset included 202,320 horses, with 19.0% of the records being censored because these horses were still actively competing in 2022. The longevity was measured based on the number of years spent in competition. The model used for the analysis of competition data was described by Dugué et al. (2021). It was based on survival analysis and included the fixed effects of region of birth, month of birth, year of performance, age at the first competition and performance level and the random effects of the sire (n = 19,225) and the maternal grandsire (n = 15,056). Heritability was estimated at 0.12.

We selected sires with the highest and lowest EBVs and sufficient reliability from those with progeny born between 2014 and 2020, allowing us to collect measurements on their offspring prior to competition between 2018 and 2022. Out of the 19,225 stallions that sired the 202,320 horses in competition, 3,722 had progeny born in France during this period. From these, 1,221 stallions either had an EBV reliability above 0.30 or had produced at least ten progeny with recorded longevity performance. Sires in the protocol were chosen from these 1,221 stallions. The average breeding value for relative risk among these 1,221 stallions was 0.83 (sd 0.15), with values ranging from 0.53 to 1.54. The mean reliability was 0.50 (sd 0.21), ranging from 0.12 to 0.98.

### 2.2 Horses

A total of 147 sires were selected from the top high and low EBVs for functional longevity among the 1,221 potential sires. The number of favorable (F) sires was 62 and unfavorable (U) sires 85. The maximum EBV for relative risk of the F sires was 0.75, the mean was 0.66 (sd: 0.05), the minimum EBV of U sires was 0.75, the mean was 0.85 (sd: 0.06).

A total of 952 horses, randomly sampled from the progeny of these sires was collected from 2018 to 2022. The number of horses sired by F sires was 590 (62% of the sample, mean of 9.5 progenies per sire, minimum 1, maximum 59), and the number of horses sired by U sires was 362 (38% of the sample, mean of 4.3 progeny per sire, minimum 1, maximum 19). Horses were born between 2014 and 2020. Blood samples were taken when the horses were aged 2 (21%), 3 (43%) and 4 (36%) years of age. There were 48% females, 21% geldings and 32% males. Data were recorded during 42 visits to the farms (combination of nearby location and date). They were mainly Selle Français (94%) or otherwise issued from a Selle Français stallion (2%) or from other European sport horse breeds (4%).

### 2.3 Power of the protocol

The analysis of the protocol was based on the idea of comparison of the mean of blood parameters between the progeny of the two groups of extreme sires. We compared the power of this statistic with that of the genetic correlation estimated in a random sample of the same size.

The power of the comparison of means between the two groups was calculated based on the expected difference, considering the actual and known difference in breeding values for functional longevity, as well as on unknown heritability of blood parameters and genetic correlation between these parameters and functional longevity. The number of horses in each group corresponds to the actual measured number.

Power of estimation of genetic correlation was calculated as the probability to declare the estimated genetic correlation different from zero, with different case of heritability of blood parameters. The standard error of the estimated genetic correlation was calculated using Tallis (1959) formulae in case of half sibs families with the actual number of sires and progeny per sire.

### 2.4 Blood analysis

Blood samples were taken at rest in the horses’ living environment. Horses had not been fed for at least 30 min and housed in a box. Five dry tubes (with coagulation activator) were collected for serum to measure biochemistry, endocrinology, proteins, bile acids, and serology. Two EDTA tubes were used to assess hematology parameters. One lithium heparin tube was collected for plasma to measure biochemistry, endocrinology (cortisol in a dry tube), CK, and vitamins. Finally, one sodium citrate tube was used to analyze coagulation by isolating platelets. Table 1 and Supplementary Material list all 59 variables, their descriptive statistics, and abbreviations. Supplementary Material provide their raw distribution before and after transformation. The variables were categorized into eight groups; hematology, liver function, kidney function, oxidative stress, cytokines, protein and endocrinology. When a parameter could be divided into components, only the total quantity and the proportional components were analyzed. For example, for proteins, the total concentration was used, along with the proportion of globulin to albumin and the various globulins relative to total proteins.

**TABLE 1:** The 59 blood variables and their descriptive statistics before transformation.

Group	Variable	Unit	Number of horses	Mean	Sd	Min**	Max**	Transformation
Bone and joint	Calcium	mg/L	934	125.7	5.5	102.8	143.3	—
	C-terminal telopeptide type II collagen	pg/mL	934	67.4	68.6	2.9	1121.4	ln
	Copper	mg/L	934	1.0	0.2	0.5	2.2	ln
	Hydroxyproline	mg/L	934	1.4	1.0	0.0	6.2	ln
	Osteocalcin	ng/mL	934	7.4	6.0	0.1	80.3	ln
	Phosphorus	mg/L	934	39.7	9.5	6.7	106.9	ln
	Vitamin D	µg/L	934	11.8	4.7	1.2	41.6	ln
	Zinc	mg/L	934	0.7	0.2	0.4	2.3	ln
Cytokine	Interleukin 10	pg/mL	934			115.6	14,400.0	discrete
	Interleukin 1	pg/mL	934			23.4		discrete
	Interleukin 2	pg/mL	934			35.2	2250.0	discrete
	Interleukin 4	pg/mL	934			16.4		discrete
	Interleukin 6	pg/mL	934			46.9	1000.0	discrete
	Interferon γ	pg/mL	934			31.3	2000.0	discrete
	Tumor necrosis factor α	pg/mL	934			7.8	1000.0	discrete
Endocrinology	Adrenocorticotropic hormone	pg/mL	916	21.7	18.0	6.1	335.0	ln
	Cortisol	nmol/L	923	98.2	37.9	27.6	281.0	—
Hematology	White blood cells	10^9^/L	952	8.8	2.0	3.7	18.0	—
	Large lymphocytes	%	952	0.7	0.3	0.0	3.0	—
	Monocytes	%	951	4.1	1.3	0.9	10.7	logit
	Basophils	%	952	0.7	0.5	0.0	3.9	logit*
	Eosinophils	%	952	2.7	1.8	0.0	13.0	logit*
	Small lymphocytes	%	952	42.6	8.0	14.0	73.2	—
	Neutrophils	%	952	49.3	8.3	18.9	81.4	—
	Red blood cells	10^12^/L	952	8.0	0.9	5.6	11.6	—
	Mean corpuscular hemoglobin concentration	g/dL	952	33.0	0.9	30.2	38.7	ln
	Mean corpuscular hemoglobin	pg	952	15.7	0.9	13.1	19.3	—
	Mean corpuscular volume	fL	952	47.6	2.7	40.1	57.2	—
	Hemoglobin	g/dL	952	12.5	1.3	9.1	17.5	—
	Hematocrit	%	952	38.0	4.3	26.6	53.5	logit
	Platelets	10^9^/L	952	155.4	39.5	21.0	370.0	—
	Red blood cells/white blood cells	10^3^	952	0.9	0.2	0.5	1.9	—
Kidney function	Creatine kinase	IU/L	952	235.0	169.8	76.0	2720.0	ln
	Creatinine	mg/L	952	12.1	1.6	7.1	19.3	—
	Urea	g/L	952	0.3	0.1	0.1	0.7	ln
Liver function	Bile acid	µmol/L	952	5.3	2.7	0.1	34.4	ln
	Total bilirubin	mg/L	952	13.7	7.1	0.2	73.0	ln
	γ-glutamyltransferase	IU/L	952	21.1	38.2	3.0	551.0	ln
	Glutamate dehydrogenase	IU/L	952	17.5	92.7	1.0	1558.0	ln
	Aspartate transaminase	IU/L	952	259.2	106.8	131.0	1374.0	ln
	Total alkaline phosphatase	UI/L	934	401.9	154.5	157.0	1791.0	ln
	Liver alkaline phosphatase	%	934	73.2	8.2	40.4	91.4	logit
	Intestinal alkaline phosphatase	%	934	11.9	4.3	4.3	33.9	logit
	Bone alkaline phosphatase	%	934	14.8	4.7	4.0	37.1	logit
Oxidative stress	Glutathione peroxidase	IU/L	934	37,267.3	16,666.0	943.0	90,815.0	—
	Glutathione peroxidase/hemoglobin	IU/g Hg	934	298.8	131.8	7.0	700.0	—
	Superoxide dismutase	IU/mL	934	258.3	65.3	82.0	559.0	—
	Superoxide dismutase/hemoglobin	IU/g Hg	934	2068.1	505.6	726.0	5375.0	ln
	Plasma lipid peroxides	µmol/L	916			7.0		discrete
	Protein carbonyls	nmol/mg Pro	914	1.4	1.2	0.0	14.0	ln
Protein	Total protein	g/L	934	64.6	6.7	30.4	88.9	—
	Fibrinogen	g/L	951	2.2	0.5	0.4	7.0	ln
	Total globulin	%	934	55.4	5.7	29.6	81.2	—
	α_1_-globulin	%	934	4.5	1.2	1.2	10.4	—
	α_2_-globulin	%	934	13.6	2.1	5.1	32.4	logit
	β_2_-globulin	%	934	7.7	3.7	1.9	30.2	logit
	β_1_-globulin	%	934	14.6	4.2	3.5	33.9	logit
	γ-globulin	%	934	15.0	2.9	4.4	26.5	—
	Serum amyloid A protein	µg/L	934			1.3	100.0	discrete

^*^: the value used for the transformation was the original value+ 0.001 to avoid problems with zero values.

^**^: For variables transformed into discrete variables, the values are the thresholds used to define the categories.

Several variables were transformed (Table 1) before further analysis. First, when more than two-thirds of the values for a variable are known only as being below or above certain thresholds (i.e., bounded values), the variable is transformed into a discrete variable with two or three categories. Specifically, the categories are: (1) values below the lower threshold, (2) values within the range between the lower and upper thresholds, and (3) values above the upper threshold. Second, variables expressed as a percentage were logit-transformed when the skewness indicator, the kurtosis indicators, and the *p*-value of the Shapiro-Wilk statistic were improved. Third, variables with distributions different from the normal distribution (*p*-value of the Shapiro-Wilk statistic < 95% or skewness > 1 or absolute value of kurtosis > 2) were ln-transformed.

In addition to blood variables, the horse’s weight was estimated using established regression on measurements: heart girth, height at withers, body length (point of shoulder to point of the croup) (Doligez and Masne, 2018). Phenotypic correlations between the variables were studied.

### 2.5 Genetic parameters

Heritability of all variables was estimated using the univariate mixed model described in Equation 1.

y=Xb+Zu+e (1)

where **y** is the vector of each blood parameter transformed variable, ***b*** is the vector of fixed effects including age effect (three levels: 2 years, 3 years, 4 years), sex effect (three levels: male, female, gelding), estimated weight effect (covariable; mean estimated weight: 526.7 kg; sd: 53.5 kg) and the visit (42 levels), ***u*** is the vector of random animal additive genetic value, $V\left(u\right)=A\sigma_{u}^{2},$ where ***A*** is the relationship matrix constructed from ancestors up to six generations (number of horses in pedigree was 10,280), ***X*** and ***Z*** are designed matrices, and e is the vector of residuals. ASReml software (Gilmour et al., 2015) was used.

The weight estimation precision was approximately ±20 kg (Doligez, 2018).

### 2.6 Analysis of the difference between blood parameters of the progeny of the two groups of sires

To analyze differences between horses’ blood parameters that could be linked to their sire’s group, we chose to use Partial Least Squares regression. The Partial Least Square regression (PLS) was performed with a logit link function to predict the group of sires (F or U) from adjusted blood parameters. In contrast to stepwise multiple regression, which suppresses variables with strong collinearity, PLS retains all variables with strong explanatory power, regardless of their relationship. The algorithm used in the plsRglm package (Bertrand et al., 2014) enabled the utilization of all the variables, even in the case of missing data, without rejecting the observations. Keeping only significant fixed effects, blood parameters were corrected according to Equation 2 and standardized according to Equation 3 in order to get adjusted blood parameters used in the PLS regression.

$$y_{c}=y-X\hat{b}$$ (2)

$$y^{*}=\frac{y_{c}-\bar{y_{c}}}{\sigma_{y_{c}}}$$ (3)

The model used for the regression is described in Equation 4.

$$g{\left(\theta \right)}_{i}=\sum_{h=1}^{H}\begin{array}{c} \left(c_{h}\sum_{j=1}^{p}w_{jh}y_{ij}^{*}\right) \end{array}$$ (4)

where ***θ*** is the probability vector, ***g*** is the *logit* link function, ***i*** is the horse, ***p*** is the number of variables, $y_{ij}^{*}$ is the adjusted and standardized variable ***j*** for horse ***i***, ***H*** is the number of components, $w_{jh}$ is the coefficient of the variable ***j*** for the component ***h***, and $c_{h}$ is the coefficient of each component ***h***. Analysis was first performed on the whole sample and a cross-validation was then performed with five randomly-chosen equal k-folds, repeated 100 times. The number of components ***H*** was chosen according to the minimum of Akaike Information Criterion (AIC) and Bayesian Information Criterion (BIC) statistical indicators over the whole sample, as well as the number of components that led to the fewest misclassified horses in the validation set during cross-validation.

## 3 Results

### 3.1 Statistical power

The genetic difference for functional longevity between the two groups was 0.68 genetic standard deviation, with a mean reliability of 0.75. With a significance level of 5%, the power of the protocol was at least 90% for traits with a heritability equal or higher than 0.20 and an absolute genetic correlation equal of higher than 0.65. For heritability of 0.30 the minimum genetic correlation needed to reach a power of 90% was 0.53 and 0.46 for a heritability of 0.40. The protocol was then expected to detect traits with medium heritability and medium genetic correlation. In comparison, a protocol based on random sample and estimated genetic correlation needed a heritability of the blood parameter of 0.30 and a genetic correlation of 0.98 to reach 90% power, and still a genetic correlation of 0.88 with a heritability of 0.40. The proposed protocol, for a range of heritability between 0.10 and 0.60 and a range of genetic correlation between 0.40 and 0.90 was on average 1.5 times more powerful than the estimation of genetic correlation (from 1.02 to 2.13).

### 3.2 Age, sex, visit and estimated weight effects

All estimated effects are presented in phenotypic standard deviation. The visit effect was always significant (*p*-value < 0.05), the standard deviation of this effect was on average 0.56.

**FIGURE 1:**
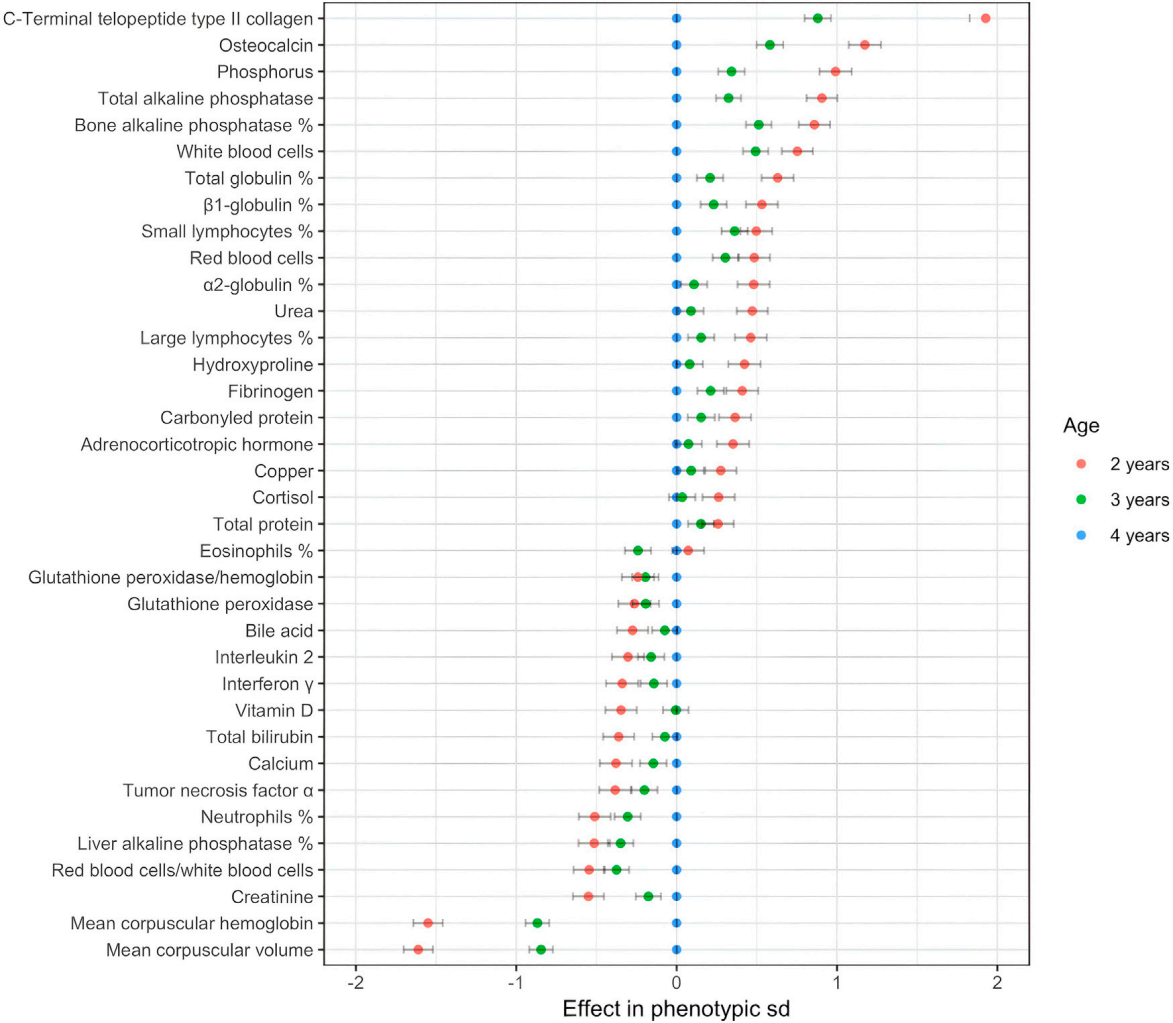
Effect of age on blood variables (only *p*-values < 0.05 are shown; reference: 4 years).

Age had a significant effect on 36 variables (Figure 1). There were 14 variables for which the absolute value of the effect of age was greater than 0.5. The estimation of the ‘3-year’ effect was consistently between the estimations of the ‘2-year’ and ‘4-year’ effects, except for the proportion of eosinophils. This pattern indicated a monotonic function. To summarize, among the 36 variables, the relevant effects are the following: the cytokine and the protein variables were higher in older horses. The ‘endocrinology’ and ‘bone and joint’ variables–except for calcium and vitamin D–were higher for young horses. The young horses had a higher red blood cell count (RBC). Mean corpuscular volume (MCV) and mean corpuscular hemoglobin (MCH) were–importantly–higher for older horses. The young horses had higher WBC, small and large lymphocyte proportions, but lower neutrophile proportion. Total ALP primarily decreases with age along with bone ALP, but liver ALP increases with age.

**FIGURE 2:**
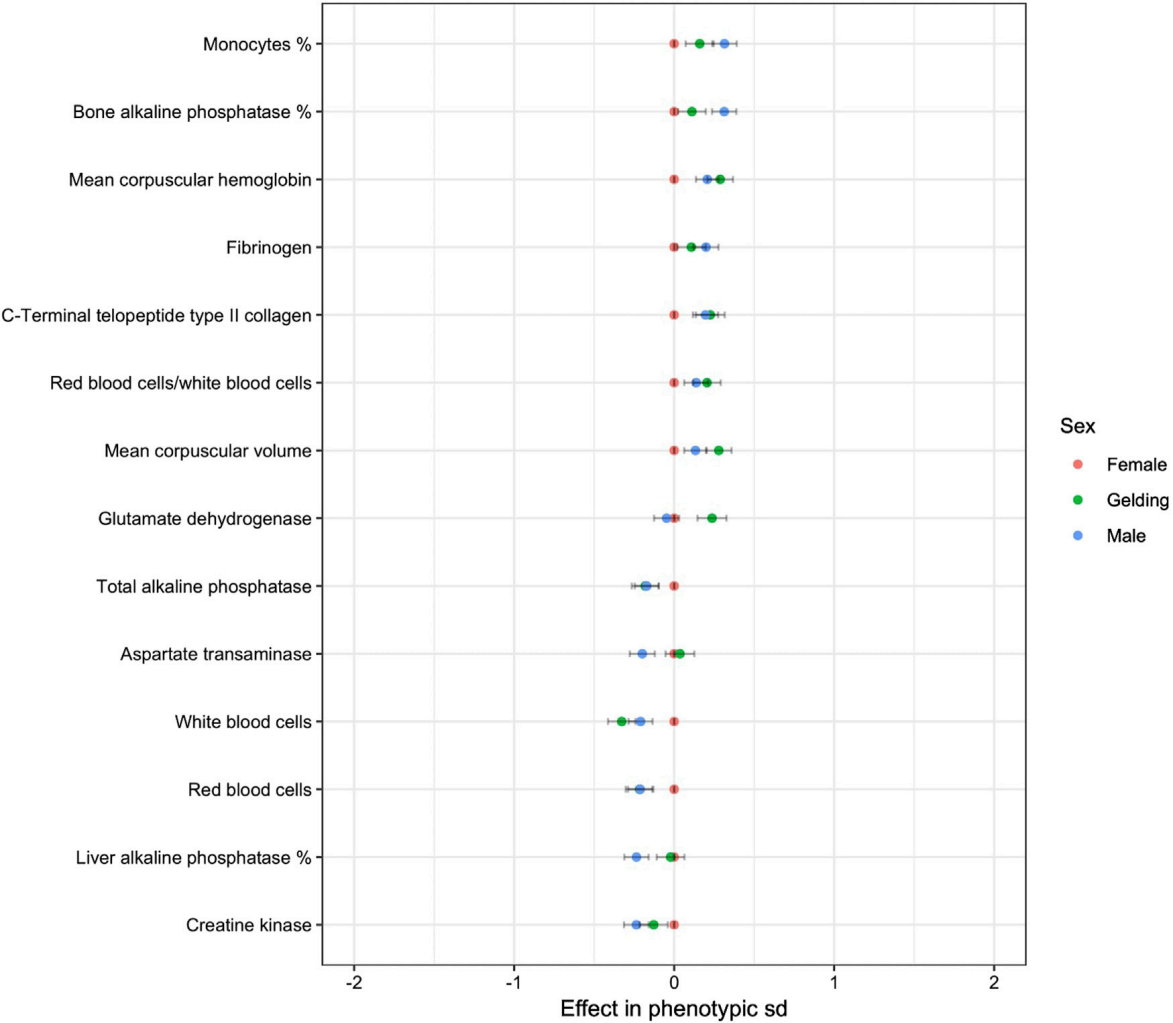
Effect of sex on blood variables (only *p*-values < 0.05 are shown. reference: Female).

Sex was found to have a significant effect on 19 variables (Figure 2). The absolute value of the effects was always lower than 0.5. Males and geldings had lower RBC but higher MCH and MCV than females. They had lower WBC but a higher proportion of monocytes. They had lower total ALP and liver ALP but higher bone ALP.

**FIGURE 3:**
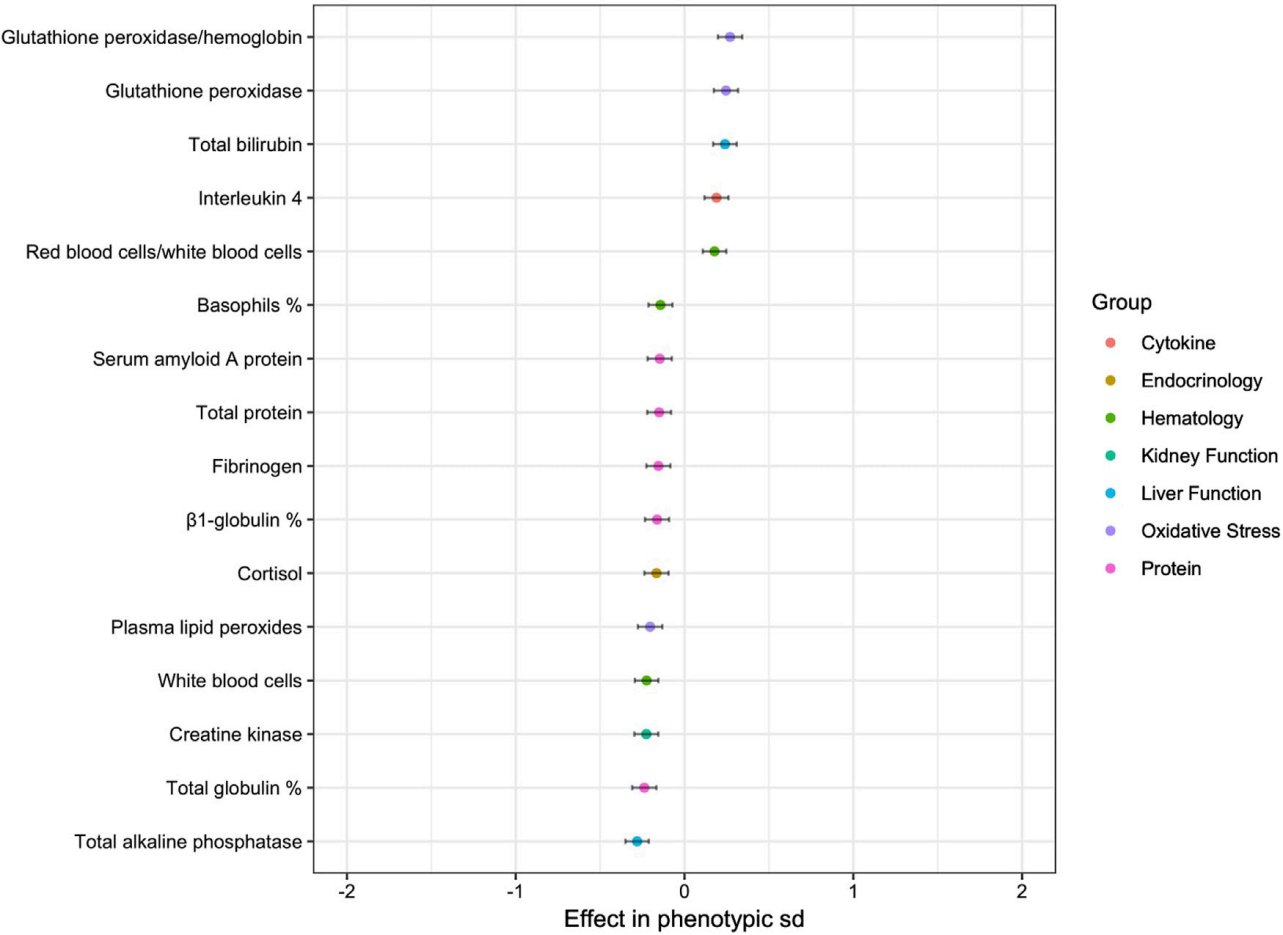
Effect of estimated weight on blood variables (for 100 kg) (only *p*-values < 0.05).

The estimated weight was found to have a significant effect on 16 variables, which are represented in Figure 3. The effects were low for a 100-kg horse, i.e., 2 sd of estimated weight: lower than 0.30 in absolute value. Among the 16 variables, estimated weight had a positive effect on proteins, creatine kinase, total ALP and white blood cells.

### 3.3 Phenotypic correlations

**FIGURE 4:**
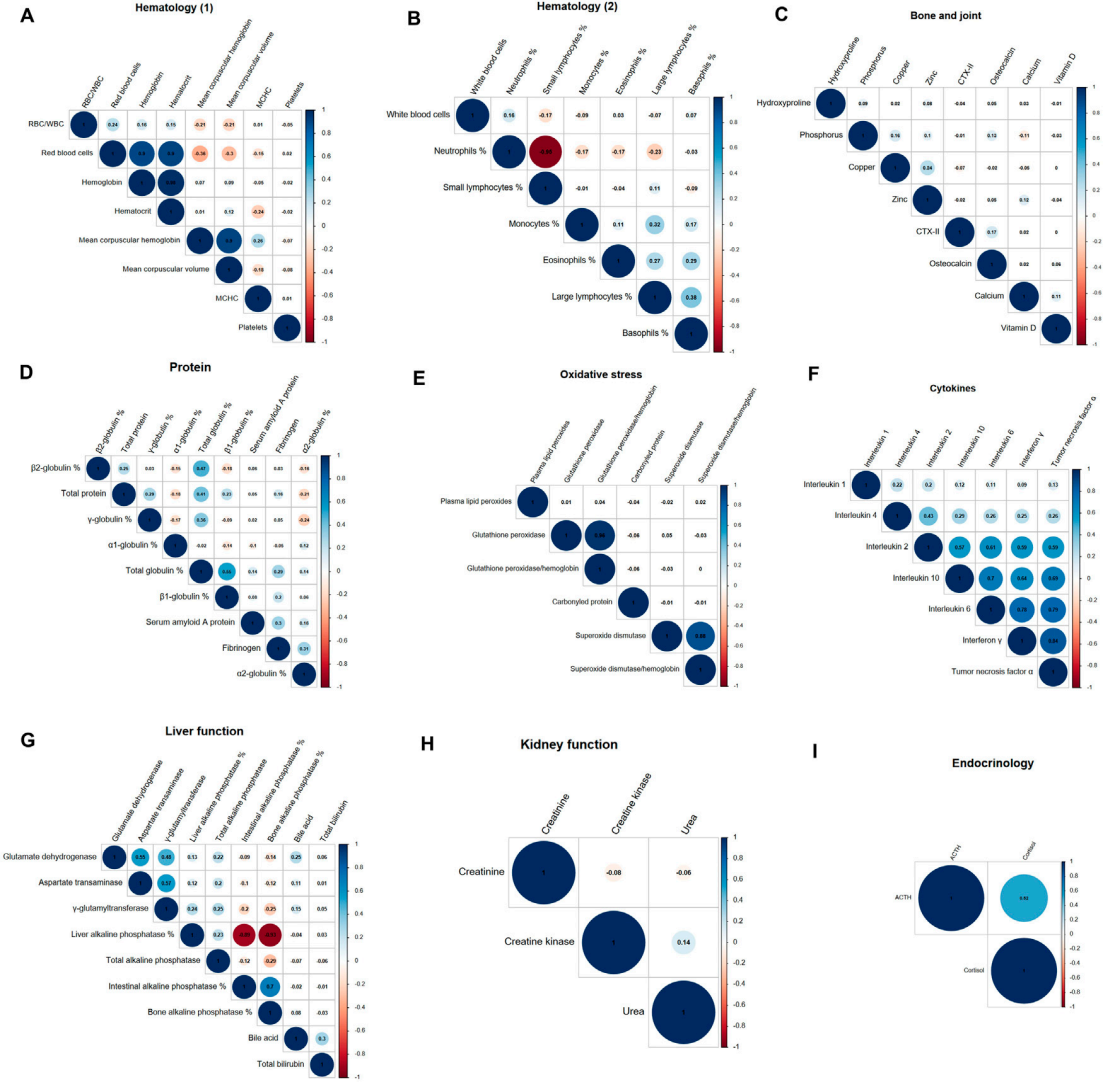
Phenotypic correlations within variable groups: Hematology **(A)**; **(B)**, bone and joint **(C)**, protein **(D)**, oxidative stress **(E)**, cytokine **(F)**, liver function **(G)**, kidney function **(H)**, endocrinology **(I)**.

The plots of the phenotypic correlations among each group of adjusted and standardized variables are given in Figure 4. Hemoglobin, hematocrit and RBC were positively correlated with one another (r > 0.90), as were MCH and MCV (r = 0.90). RBC was negatively correlated with MCH and MCV (r < −0.30). The proportion of neutrophils was negatively correlated with the proportions of small lymphocytes (r = −0.95) and large lymphocytes to a lesser extent (r = −0.23). Proportions of eosinophils, basophiles and monocytes were less positively correlated with the proportion of large lymphocytes (0.27 < r < 0.39). WBC count was not correlated with the proportions of the variables that constitute WBC but was positively correlated with hematocrit, hemoglobin and RBC (0.33 < r < 0.35).

The proportion of total globulin was positively correlated with the proportions of β2-globulin, β1-globulin and γ-globulin, and total protein (0.36 < r < 0.55). To a lesser degree, the proportion of β2-globulin was negatively correlated with the proportions of β1- globulin, α1-globulin and α2- globulin (−0.18 < r < −0.15).

Among the cytokines, interleukin 2, 10, 6, interferon γ and tumor necrosis factor α were positively correlated with one another (0.57 < r < 0.84).

The proportion of liver ALP was highly negatively correlated with the proportions of intestinal ALP and bone ALP (r < −0.89). Those two were positively correlated (r = 0.70). Glutamate dehydrogenase (GLDH), aspartate transaminase (AST) and γ-glutamyltransferase (GGT) were positively correlated with one another (r > 0.48).

There were no phenotypic correlations among the ‘bone and joint’ group or in the ‘kidney function’ group. In the ‘oxidative stress’ group, the only significant phenotypic correlations were between ratios and their constituent variables.

There were no high phenotypic correlations between variables across different functions. Inter-function correlations were the following: bone ALP was positively correlated with osteocalcin and C-Terminal telopeptide type II collagen; and neutrophil proportion was negatively correlated with glutamate dehydrogenase and aspartate transaminase (r < −0.24). Calcium was slightly negatively correlated with the proportion of total globulin (r = 0.30). Total protein, zinc, creatinine and superoxide dismutase were slightly positively correlated with RBC, hemoglobin and hematocrit (r >0.17). γ-glutamyltransferase was negatively correlated with intestinal and bone ALP (r = −0.20 and r = −0.25). Total protein was positively slightly correlated with WBC (r = 0.23). Glutathione peroxidase (GPx) was negatively correlated with the proportion of β1-globulin (r = −0.26).

### 3.4 Heritability

Estimates of heritability are given in Table 2. Some 75% of heritabilities was greater than 0.20% and 30% greater than 0.50. The standard errors ranged from 0.06 (low heritabilities) to 0.13. For the ‘bone and joint’ group, heritability of copper and vitamin D were around 0.5 and the others were lower than 0.25. For the ‘cytokine’ and ‘endocrinology’ groups, all the heritability estimates were lower than 0.40. In the ‘hematology’ group, heritability ranged from 0.16 to 0.92. The two higher heritability estimates with a large difference with the others were MCH (0.90) and MCV (0.92), and their ratio (MCHC) was still high (0.43). Heritability estimates of the number of WBC per mm^3^ and the number of RBC were high (0.55 and 0.47), as was their ratio (0.52) and number of platelets (0.54). The heritability of variables describing the composition of WBC in percentage was lower than 0.40. For the ‘kidney function’ group, heritability ranged from 0.33 to 0.56. For the ‘liver function’ group, apart from GLDH (0.08) and AST (0.17), the variables were highly heritable, with the highest value for total ALP (0.68). In the ‘oxidative stress’ group, the values ranged between 0.00 and 0.60. The highest values were the heritability of superoxide dismutase/hemoglobin (0.54) and the heritability of superoxide dismutase (0.60). For the ‘protein’ group, the values were between 0.12 and 0.57. The highest values were the heritability of total protein (0.47) and the proportion of γ-globulin (0.57).

**TABLE 2:** Heritability of the blood variables.

Group	Variable	h^2^	Se
Bone and joint	Calcium	0.22	0.10
	C-terminal telopeptide type II collagen	0.22	0.10
	Copper	0.50	0.13
	Hydroxyproline	0.19	0.11
	Osteocalcin	0.13	0.08
	Phosphorus	0.24	0.10
	Vitamin D	0.54	0.13
	Zinc	0.12	0.08
Cytokine	Interleukin 10	0.23	0.11
	Interleukin 1	NE	NE
	Interleukin 2	0.26	0.11
	Interleukin 4	0.40	0.12
	Interleukin 6	0.09	0.08
	Interferon γ	0.23	0.11
	Tumor necrosis factor α	0.36	0.12
Endocrinology	Adrenocorticotropic hormone	0.17	0.09
	Cortisol	0.36	0.11
Hematology	White blood cells	0.55	0.13
	Large lymphocytes %	0.16	0.09
	Monocytes %	0.39	0.12
	Basophils %	0.30	0.11
	Eosinophils %	0.26	0.10
	Small lymphocytes %	0.22	0.09
	Neutrophils %	0.25	0.10
	Red blood cells	0.47	0.12
	Mean corpuscular hemoglobin concentration	0.43	0.11
	Mean corpuscular hemoglobin	0.90	0.11
	Mean corpuscular volume	0.92	0.11
	Hemoglobin	0.34	0.11
	Hematocrit	0.32	0.11
	Platelets	0.54	0.12
	Red blood cells/White blood cells	0.52	0.12
Kidney function	Creatine kinase	0.33	0.11
	Creatinine	0.56	0.13
	Urea	0.52	0.13
Liver function	Bile acid	0.52	0.13
	Bilirubin total	0.53	0.12
	γ-glutamyltransferase	0.30	0.10
	Glutamate dehydrogenase	0.08	0.06
	Aspartate transaminase	0.17	0.08
	Total Alkaline phosphatase	0.68	0.12
	Liver Alkaline phosphatase %	0.53	0.13
	Intestinal Alkaline phosphatase %	0.52	0.13
	Bone Alkaline phosphatase %	0.53	0.12
Oxidative stress	Glutathione peroxidase	0.13	0.09
	Glutathione peroxidase/hemoglobin	0.27	0.12
	Superoxide dismutase	0.60	0.11
	Superoxide dismutase/hemoglobin	0.54	0.11
	Plasma lipid peroxides	0.10	0.06
	Protein carbonyls	0.00	0.00
Protein	Total protein	0.47	0.13
	Fibrinogen	0.13	0.08
	Total globulin	0.28	0.11
	α1-globulin %	0.16	0.08
	α2-globulin %	0.26	0.10
	β2-globulin %	0.32	0.11
	β1-globulin %	0.36	0.12
	γ-globulin %	0.57	0.12
	Serum amyloid A protein	0.12	0.08

### 3.5 Blood markers and functional longevity

The different criteria used to select the number of components included in the model are shown in Table 3. The minimum of AIC was obtained for four components, the minimum of BIC was obtained for three components, and the minimum of misclassified horses was obtained for six components. Cross-validation on the basis of the number of misclassified horses in the validation sample proposed one component. We chose a compromise between these criteria by setting the number of components at two.

**TABLE 3:** Statistical criteria (AIC, BIC, number of misclassified horses) for the entire dataset, and cross-validation results: number of replicates (out of 100) selecting each component based on the number of misclassified horses in the validation set (using 5-fold cross-validation).

Number of components	AIC	BIC	Misclassified	Number of replicates selecting component (out of 100)
1	1203	1213	342	88
2	1182	1197	335	0
3	1174	1193	330	4
4	1173	1198	329	2
5	1174	1203	316	2
6	1176	1208	309	1
7	1175	1214	316	2
8	1176	1220	313	1

The PLS logistic regression with two components showed that 16 variables had a significant effect in distinguishing the group of sires based on their longevity EBV. The regression coefficients and associated *p*-values are given in Table 3. The AUC was 67% and there were 330 horses misclassified among the 952.

**FIGURE 5:**
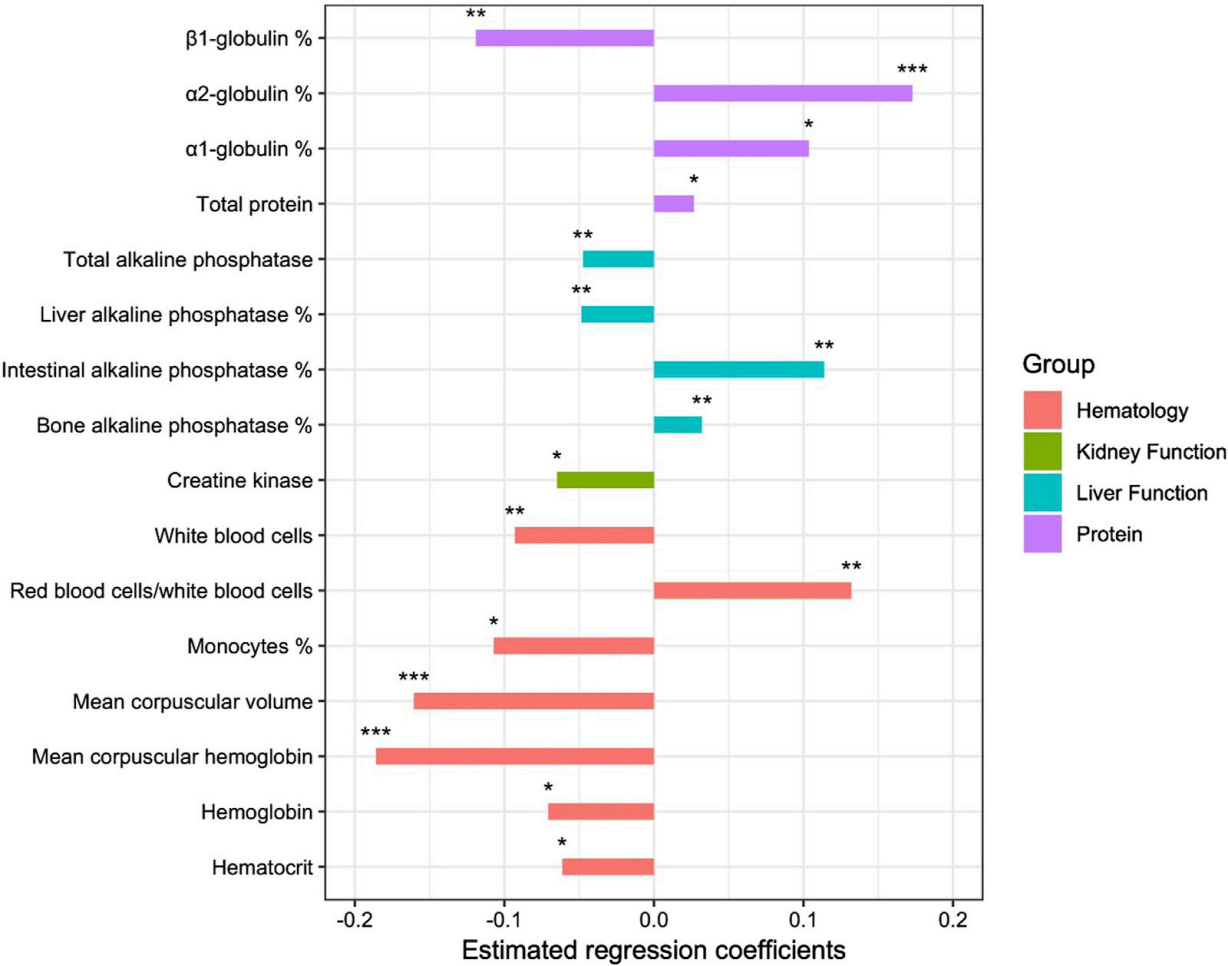
Estimated regression coefficients of the effect of the variables on probability to belong to the favorable group of sires for functional longevity (*p-value < 0.05; **p-value < 0.01; ***p-value < 0.001).

We found 16 variables that had a significant effect on the longevity group to which the horse belonged (Figure 5). Some had a negative effect on longevity, i.e., they favored membership in the group with low functional longevity EBV; these were the proportions of α1-globulin and α2-globulin, total protein, RBC/WBC ratio as well as bone and intestinal ALP proportions. Others favored membership in the group with good functional longevity EBV; the proportion of β1-globulin, hemoglobin, hematocrit, WBC, the proportion of monocytes, MCV, MCH, creatine kinase, total ALP and the proportion of liver ALP. No variables in the ‘bone and joint’, ‘oxidative function’ or ‘endocrinology’ groups had any significant effect on longevity.

## 4 Discussion

We found significant effects of age, sex, estimated weight and visit on several variables, which confirms the relevance of their inclusion as fixed effects in the final model. There has been a lack of consensus in the literature regarding these fixed effects, making it difficult to compare our findings. Moreover, our study specifically focused on horses aged 2 to 4 years—a critical growth period—while most previous research has examined either very early growth stages or the effects of aging. However, age-related findings in the literature, such as a decrease in WBC with age (Satué et al., 2020), are consistent with our results. We confirmed the global negative impact of age on total ALP and the bone ALP fraction, and a positive impact on the liver ALP fraction (Thoren-Tolling, 1988). Our findings on RBC, MCV and MCH in young horses were consistent with Satué et al. (2020).

Regarding sex, our study found no effect on hemoglobin, contradicting previous findings (Pađen et al., 2014; Prvanović Babić et al., 2019; Satué et al., 2020). Males had lower red and white blood cell counts in our study, only partially consistent with Satué et al. (2020). Our results for creatine kinase and total ALP were consistent with Pađen et al. (2014), but we found no sex effect on other variables they identified.

To summarize, age and sex effects on hematological values varied widely, probably influenced by the diversity of breeds in studies (Prvanović Babić et al., 2019). Moreover, our study provided more precise effect identification due to the larger sample size.

Heritability estimates were potentially overestimated because the sample was selected from the progeny of extreme stallions for longevity breeding values. This is the case if the variable was actually related to longevity. This may explain, for example, the very high heritability estimates for MCH and MCV (>0.90). Heritability of all variables with a significant effect on the group of stallions was higher than 0.30, except for percentage of α1-globulin (0.16) and α2-globulin (0.26). The only study found with a sufficiently large population to estimate heritability for blood variables in horses was Norton et al. (2019), which estimated heritability with a genomic relationship for Welsh ponies and Morgan horses. The only common variable with our study was ACTH, which was only significant for Welsh ponies (*p*-value = 0.05), with a value of h^2^ = 0.305. Our result was h^2^ = 0.17. This difference might be the effect of the breed, that is metabolically different from Selle Français horses. We calculated heritability estimates for many traits that had not been studied before. Many blood variables were strongly to moderately heritable, even those not linked to longevity. For traits linked to the group of sires, this means that they are potentially selectable characteristics.

A Partial Least Square Regression was used to facilitate the interpretation of the regression. The optimum number of components for the PLS logistic regressions differed among the statistical criteria. Cross-validation yielded a lower number of components compared to criteria calculated from the whole sample. To mitigate the risk of overfitting, we chose to closely adhere to the cross-validation result and retained two components. The logistic regression revealed that 16 variables significantly influenced the probability of belonging to either the favorable or unfavorable group of sires selected based on their breeding values for functional longevity. The distinguishing factor between the two progeny groups was their own breeding value for functional longevity, inherited from their sire. In consequence, the blood variables identified are directly associated with the genetic value of the horse for functional longevity. These effects will henceforth be referred to as having a positive or negative effect on functional longevity genetic values.

The variables found to have the most significant negative effect on functional longevity genetic value were MCH and MCV, which were highly phenotypically correlated and very heritable (over 0.90) and, therefore, of genetic interest. MCV, which is lower for mature erythrocytes compared to immature ones (reticulocytes), tends to increase during macrocytic anemia induced by hemorrhage or sever hemolytic disorders (Radin et al., 1986; Satué et al., 2014), along with MCH. Muñoz et al. (2008) highlighted contradictions among various authors regarding the relationship between these parameters and equine physical exertion. Hemoglobin also had a negative effect on the longevity genetic value; an increase in MCH that may result from an increase in MCV naturally leads to an increase in hemoglobin, partially explaining the negative effect of hemoglobin. Hemoglobin levels tend to be lower in trained horses (Muñoz et al., 1999) with a lower increase during exercise. Moreover, we found a negative effect of hematocrit on the longevity genetic value. Hematocrit increases in case of dehydration (Geor and McCutcheon, 1998). Horses with lower hydration levels may experience premature reductions in stroke volume and cardiac output, indicating early signs of fatigue and potentially reduced resistance to physical effort, leading to premature retirement. Moreover, while optimal levels enhance oxygen transport capacity, deviations above or below this optimum degrade oxygen transport (Birchard, 1997). An increased hematocrit level that exceeds the optimal range would be less favorable for functional longevity than a decreased one. The two important points to focus on to favorize better functional longevity seem to be a good hydration status along with an absence of macrocyte anemia. In particular, prior studies predominantly focused on horses in training, whereas we assessed young horses at rest, exhibiting values within normal ranges. Creatine kinase had a moderate negative effect on the longevity genetic value and was moderately heritable (0.33). Its plasma concentration is usually low because it is predominantly found in cardiac and skeletal muscles (Thornton and Lohni, 1979). An increase in CK in horses indicates muscle damage, such as skeletal muscle injuries (Octura et al., 2014), revealing a sensitivity to muscle soreness, potentially shortening their sports career. CK levels in athlete horses’ blood fluctuate according to a circadian rhythm (Piccione et al., 2009), adding noise to our statistical analysis. WBC count increases in horses during inflammatory reaction and is used to diagnose various diseases such as laminitis, hypersensitivities, infections, sepsis (Schnabel et al., 2015) – as well as to assess the horse’s immunological status. **S**tress also significantly affects WBC count; the reaction to stress has been described as an inflammatory-like response by several authors (Arfuso et al., 2023; Cywińska et al., 2015). Our data showed no abnormal WBC values, indicating no illness or stress at the time of sampling. However, slight positive variations can signal mild stress or light inflammation, suggesting a sensitivity, that could lead to early signs of fatigue over time, potentially explaining the negative effect of WBC. However, the positive effect of α2-globulin proportion, an acute phase protein (APP) during inflammation and whose activity is link to that of WBC (Abeni et al., 2013; Hultén et al., 2002) was not expected. Total protein had a positive effect on functional longevity genetic value; but the wide diversity of proteins complicates the interpretation, although an elevation in total protein levels might raise concerns from a clinical standpoint. In addition to that, the proportion of monocytes–part of the innate immune system -had a negative effect on functional longevity genetic value. WBC count, total protein, α2-globulin and monocyte proportion were moderately to highly heritable (above 0.26) and warrant further study, especially for WBC (h^2^ = 0.55). The negative effect of the RBC/WBC ratio on longevity genetic value might be due to the negative effect of WBC which has a significant effect was phenotypically highly correlated with RBC/WBC (−0.78).

β1-globulin and α1-globulin had an effect on functional longevity genetic value, but there was little in the literature about their precise function, making it difficult to interpret these results. The second is suspected to be a sign of gastrointestinal inflammation (Abeni et al., 2013), which is incoherent with the positive effect we found. However, β1-globulin had a negative effect on longevity genetic value, and its increase in blood is generally a sign of inflammation, pathology, liver disorders (Foreman, 2023), or intestinal parasitism (Kawsar, 2019).

The different types of alkaline phosphatase–that are liver enzymes–had various effects on functional longevity genetic value. Bone and liver ALP proportions had opposite effects although they both belong to the tissue non-specific alkaline phosphatases (Goldstein et al., 1980; Hoffmann et al., 1983). Little is known about these alkaline phosphatases. The proportion of bone ALP, important in the bone mineralization process in mammals and whose increase is mainly the sign of osteoblast activity (Sharma et al., 2014) had a positive effect. Liver ALP, which often reveals chronic inflammation disease, chronic respiratory infection, toxic or inflammatory reaction, reaction to training, stress (Thoren-Tolling, 1988) or bile flow obstruction (Bellier, 2010), had a negative effect on longevity genetic value. A horse with a higher liver ALP fraction might be more sensitive and more likely to develop such illnesses. Intestinal ALP proportion had a significant positive effect on functional longevity genetic value. In mammals, it has several key biological functions: it controls intestinal lipid absorption, regulates extracellular pH, protects intestine against inflammation, notably by reducing the toxicity of Gram + bacteria (Lallès, 2014; 2010a; 2010b). Although intestinal inflammation has been shown to significantly increase ALP concentration in mice (Sánchez De Medina et al., 2004), ALP secreted was not necessarily intestinal ALP. Because of the slight positive phenotypic correlation of total ALP and liver ALP fraction, an increase in total ALP in the case of inflammation might be due to an increase in liver ALP. It is genetically interesting to observe the high heritability of total ALP (0.68), along with the proportions of its components: 0.53 for bone ALP and liver ALP; and 0.52 for intestinal ALP for horses measured at rest. Moreover, these results are coherent with the negative phenotypic correlation between liver ALP proportion and both bone ALP and intestinal ALP proportions, as well as the positive phenotypic correlation between the latter two.

No oxidant or antioxidant molecule had a significant effect on functional longevity genetic value, nor did any of the cytokines. Cytokines, which are almost undetectable in healthy horse blood under normal conditions, are very important in inflammatory and infection reactions since they stimulate WBC production and APP when needed (Schnabel et al., 2015; Victoria Carapeto et al., 2006). Studying a potential effect of cytokines on longevity might require an experiment with an induced inflammation process. However, since these variables have low heritability compared with others (70% lower than 0.26, 30% between 0.26 and 0.40), understanding their role and the absence of a link with functional longevity seems less important than other variables. Neither hormone (ACTH and cortisol) had an effect on longevity genetic value. Both ACTH and cortisol are signs of Cushing’s Disease (Perkins et al., 2002), and cortisol is an indicator of stress (Casella et al., 2016; Sikorska et al., 2023).

This study and its results could be used to predict genetic value for functional longevity on the basis of blood parameters measured early in a horse’s life, thereby increasing the reliability of functional longevity EBVs at age of selection. Combining these results with other measured parameters analyzed using the same protocol, such as infrared images, morphology, gait characteristics, behavioral traits, physiological parameters and body composition via bioelectrical impedance analysis that are currently under investigation, could provide more comprehensive information and improve the prediction of a young horse’s functional longevity genetic value.

Since very few studies have focused on measuring biochemical and hematological parameters in young horses at rest before their entry into competition, further research is needed to confirm these results. Greater knowledge about the precise biological functions and consequences of certain blood parameters, e.g., globulins, could improve the accuracy of interpretations. To check that the characteristics of the blood variables highlighted as favoring longevity do not have a deleterious effect on performance, these parameters need to be measured on a representative sample of horses according to their performances.

## 5 Conclusion

To conclude, we identified 16 blood parameters genetically associated with functional longevity in show jumping horses, encompassing hematology, kidney and liver functions and proteins. These results highlight the fact that a lower inflammatory status, better hydration and reduced macrocyte anemia, within the normal physiological range, are important for promoting better genetic values for functional longevity. Among the most relevant parameters, lower mean corpuscular volume, lower mean corpuscular hemoglobin, lower hematocrit, lower hemoglobin, lower number of white blood cells, lower total and percentage of liver alkaline phosphatase but higher bone and intestinal fraction, higher α2-globulin and lower β1-globulin proportions were associated with a greater likelihood of belonging to the favorable group of sires for longevity. These parameters seemed heritable, easy to measure at rest in young horses and accessible to all horse owners, making them potentially valuable for early genetic evaluation of functional longevity in show jumping horses. However, before implementing these findings, we need to further explore the biological explanations and connect these parameters to the other traits measured during the protocol. In addition, we need to check heritability, quantify genetic correlation and assess the impact on performance level in an independent random sample for the blood parameters found.

## Data Availability

The datasets presented in this study can be found in online repositories. The names of the repository/repositories and accession number(s) can be found below: https://zenodo.org/records/12531357?token&equals;eyJhbGciOiJIUzUxMiJ9.eyJpZCI6IjEyZWMyMTM4LWEwZTctNGNmZi1hMTMyLWNhZjcxZDg4NzYwYyIsImRhdGEiOnt9LCJyYW5kb20iOiI0ZGQzMWI2ZWY2ZWVmN2JiMzZlMWIxYTk1MWJmMGMyNCJ9.GL3qiTqEl_ls8Lq_V7Rjbkv6E_R5e90cq5V6mhTyEwqWbh4WPKxsr5M2-QQYW45RLfMqLpLunxprR_hC11bKDw.
