# Supplementary figures and images for: Comparison of blood parameters in two genetically different groups of horses for functional longevity in show jumping
Source: Front Genet. 2024 Oct 29;15:1455790. doi: 10.3389/fgene.2024.1455790 (PMC11554460; doi:10.3389/fgene.2024.1455790)

Distribution of the variables after transformation

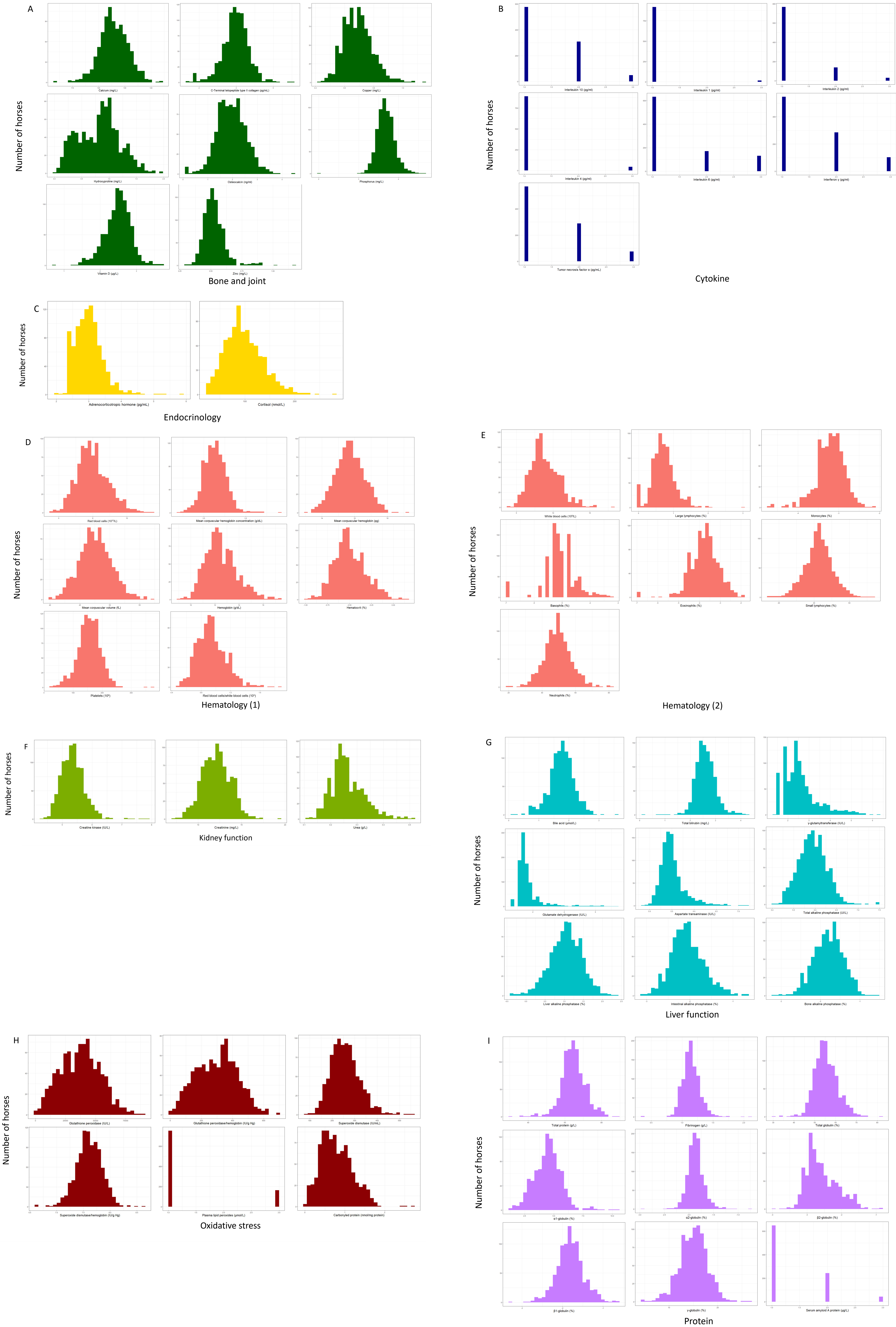

Supplement: Supplementary file 1 [file DataSheet2.PDF]

Distribution of the variables before transformation

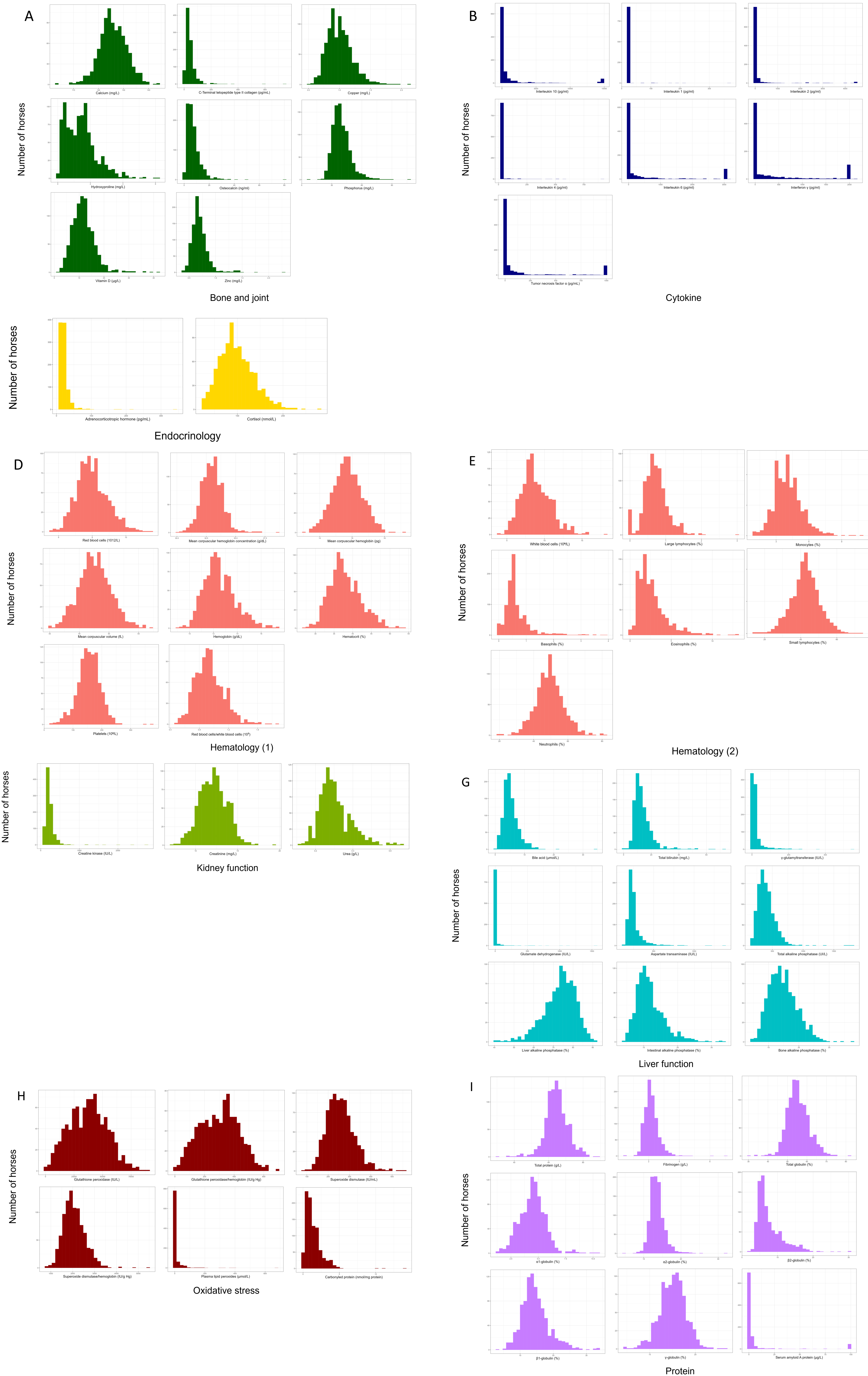

Supplement: Supplementary file 2 [file DataSheet1.PDF]
